# Characterization of the Chemical Composition, Cytotoxicity, and Metabolomic Effects of PM_2.5_ in a Plateau City, China

**DOI:** 10.3390/toxics13090729

**Published:** 2025-08-29

**Authors:** Mengying Li, Lijuan Qi, Xinyi Xu, Rong Zhao, Xiaotong Wang, Yanhui Ha, Zhe Lin, Sujin Lu, Rong Chen, Junchao Zhao

**Affiliations:** 1 College of Ecological and Environmental Engineering, Qinghai University, Xining 810016, China; 18997169373@163.com (M.L.);; 2 Key Laboratory of Beijing on Regional Air Pollution Control, Beijing University of Technology, Beijing 100124, China; 3 Key Laboratory of Vehicle Emission Control and Simulation of Ministry of Ecology and Environment, Vehicle Emission Control Center, Chinese Research Academy of Environmental Sciences, Beijing 100124, China

**Keywords:** PM_2.5_, A549 cells, plateau city, cytotoxicity, metabolomics

## Abstract

The health impacts of atmospheric fine particulate matter (PM_2.5_) in plateau regions have attracted concerns, along with local population growth and rapid urbanization. This study collected PM_2.5_ samples at summer and winter in Xining, a city located in the northeastern Tibetan Plateau. The chemical composition of PM_2.5_ and its cytotoxicity on human lung epithelial cells (A549) are characterized, and composition–cytotoxicity correlation is discussed. The toxic mechanisms of PM_2.5_ in different seasons were further investigated through metabolomic analysis using high-resolution mass spectrometry. The average PM_2.5_ mass concentration in Xining during winter was 2.10 times higher than that during summer. The carbonaceous components in PM_2.5_ were dominated by OC, while the main water-soluble ions were SO_4_^2−^, NO_3_^−^, and NH_4_^+^, with Mg, Al, Fe, and Ca also present in high concentrations in metal elements. LDH and ROS emerged as the most PM_2.5_-affected toxicity indices in summer (34.59 ± 4.86 ng/L, 1.19× control) and winter (8.62 ± 1.25 ng/mL, 1.77× control), respectively. OC, Cl^−^, F^−^, Sn, Cr, SO_4_^2−^, Pb, Zn, Mg, NO_3_^−^, and NH_4_^+^ may synergistically exacerbate oxidative stress and inflammatory responses on A549 cells in Xining. Furthermore, glutathione metabolism, amino acid metabolism, and sphingolipid metabolism were identified as key pathways influencing cellular oxidation and inflammation. Thimonacic, 9-(2,3-dihydroxypropoxy)-9-oxononanoic acid, and hypoxanthine were common metabolites in both seasons. Our findings greatly enhance the understanding of health risks associated with PM_2.5_ in the plateau city.

## 1. Introduction

Atmospheric fine particulate matter (PM_2.5_) pollution is one of the crucial factors threatening the ecosystem, and its impact on human health has received widespread attention [1,2]. PMs across different size ranges have been shown to pose various health risks, particularly fine particulate matter (PM_2.5_), which is characterized by a long atmospheric retention time and extensive transport pathways [3]. This allows harmful substances to adsorb onto its surface, facilitating their entry into the human body via respiratory and circulatory processes, potentially leading to a range of diseases [4]. Furthermore, the metallic elements in PM_2.5_ can be absorbed and accumulated by vegetables, subsequently entering the human food chain, which also contributes to an increase in potential health risks for humans [5]. Epidemiological studies have linked severe health outcomes to PM exposure, including morbidity and mortality [6]. Long-term exposure to PM_2.5_ increased the risks of respiratory illnesses, lung cancer, and cardiovascular diseases [7,8]. Toxicological studies have found that the inhalation of PM_2.5_ had a significant effect on cell membranes, cellular oxidative stress, and inflammatory damage, which in turn led to alterations in genetic material, ultimately leading to cancers and hereditary disorders, among other outcomes [9].

The chemical compositions and concentrations of PM_2.5_ play a crucial role in its cytotoxicity [10]. Oxidative stress and inflammatory responses were the primary toxic reactions of PM_2.5_ to induce lung diseases [11]. Heavy metal ions, such as Zn, V, As, and Cu, enriched in PM_2.5_, damaged alveolar cells and promoted the generation of reactive oxygen species (ROS), thereby inhibiting cellular activities [12]. The concentrations of Cr, As, Cu, Fe, Mn, Ni, and Zn in PM_2.5_ were closely associated with the PM_2.5_-induced release of the pro-inflammatory factor tumor necrosis factor (TNF-α) from human lung epithelial cells (A549) [13]. Additionally, Mn, Cu, Cd, and Pb contributed significantly to decreasing cell viability, membrane injury indicated by the release of lactic dehydrogenase (LDH), and ROS production [14]. Among the water-soluble ions, SO_4_^2−^ and NH_4_^+^ were significantly correlated with ROS production [15]. The increase of elemental carbon (EC) exhibited a significant positive correlation with intracellular DNA damage markers [16]. At the same time, the cytotoxicity of PM_2.5_ varies across different seasons. The cytotoxicity indexes were significantly higher in fall and winter than in spring and summer in some cities [17]. It has also been shown that PM_2.5_ induced a significant increase in ROS production in macrophages during summer and resulted in more pronounced changes in cellular activity compared to winter in Chapel Hill, North Carolina [18].

The toxicological effects induced by PM_2.5_ have the potentially to disrupt complex metabolite pathways and affect molecular targets. Previous studies have provided evidence that differential changes in cellular metabolite levels and alterations in metabolic pathways were closely linked to PM_2.5_ exposure [19]. For instance, elevated levels of acetoacetic acid and 3-hydroxybutyric acid were detected in the serum of mice exposed to PM_2.5_ [20]. PM_2.5_ exposure similarly disrupted the tricarboxylic acid cycle, amino acid metabolism, and glutathione metabolism in A549 cells [18].

Xining, situated in the northeastern Qinghai-Tibet Plateau with an altitude of 2261 m, is the largest and most populous city in the region in terms of economic scale and population [21]. The main emission source in northwest China was residential combustion, accounting for 34.4% of emissions and presenting more toxicity per unit mass, despite having a low emission intensity [22]. The prevalence of asthma among highland populations was found to increase with higher concentrations of PM exposure [23]. Meanwhile, cold weather and elevated PM_2.5_ levels were significantly correlated with the prevalence of respiratory diseases in a high-altitude population [24]. However, the cytotoxic effects and underlying mechanism of PM_2.5_ in a plateau city are still unknown. In this study, we determined the chemical compositions of PM_2.5_ collected in Xining, conducted in vitro toxicity experiments on A549 cells, and applied untargeted metabolomics to investigate the toxicity mechanisms. This research will provide a scientific basis for assessing health risks and formulating protection measures on PM_2.5_ pollution in the plateau cities.

## 2. Materials and Methods

### 2.1. Study Area and PM_2.5_ Sampling

**Figure 1:**
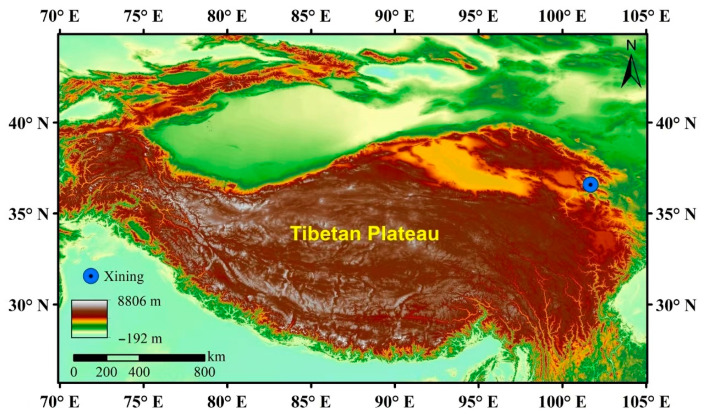
Overview of the study area.

PM_2.5_ samples were collected in June 2023 and December 2023 on the roof of the teaching building in Qinghai University in Xining (101°44′ E, 36°40′ N), shown in Figure 1. There were no obvious emission sources around the sampling site. The 12 h daily PM_2.5_ samples were collected on quartz microfiber filters (90 × 90 mm, Whatman, Piscataway Twp, NJ, USA) using medium-volume (100 L·min^−1^) air samplers. A micro air quality monitoring station was established at the site to measure the concentrations of conventional pollutants. Prior to sampling, the quartz filters were baked in a muffle furnace at 450 °C for 4 h to eliminate potential organic contaminants. After sampling, the filters were balanced at constant temperature and humidity for 24 h, then weighed again and preserved in a fridge at −20 °C for the following chemical analyses and toxicity tests.

### 2.2. PM_2.5_ Chemical Composition Analysis

The organic carbon (OC) and EC in PM_2.5_ samples were determined by a DRI-2015 OC/EC analyzer (Magee, West Hartford, CT, USA). For water-soluble components, filter pieces immersed in ultrasonic water were sonicated for 1 h in a pre-cooled ultrasonic cleaner, and then the extraction was filtered by 0.45 μm pore-size membranes to obtain the solution. The ion chromatography tool Dionex ICS-5000 (Thermo Fisher Scientific, Waltham, MA, USA) was used to measure major cations (Na+, NH_4_^+^, K^+^, Ca^2+^, Mg^2+^), and Dionex ICS-1100 (Thermo Fisher Scientific, USA) was utilized to analyze other cations (F^−^, Cl^−^, SO_4_^2−^, NO_3_^−^). For the contents of PM_2.5_-bound metals, 10 mL of mixed nitric acid–hydrochloric acid solution was added to the PM_2.5_ samples, which was digested by a microwave ablator (Milestone, Eden Prairie, MN, USA), and analyzed using an inductively coupled plasma mass spectrometer (ICP-MS, Thermo Fisher, USA) to determine the following elements: Li, Mg, Al, P, Ca, Ti, V, Cr, Mn, Fe, Co, Ni, Cu, Zn, Ga, As, Rb, Sr, Mo, Sn, Sb, Ba, Pb, and Bi.

### 2.3. Preparation of PM_2.5_ Suspension for Cell Exposure

The collected PM_2.5_ filter was cut and immersed in 100 mL of ultrapure water, followed by a low-temperature ultrasonic vibration for 1 h. After that, the PM_2.5_ suspension was filtered through eight layers of gauze; the filtrate was then frozen at −20 °C and lyophilized to obtain PM_2.5_ powder using a freeze dryer (Shanghai Ailang Instrument, Shanghai, China). Finally, based on particle mass, sterile PBS and F12K medium (Shanghai Fuheng Biotechnology, Shanghai, China) were successively added and diluted to 240 mg/L PM_2.5_ suspension. The determination of the PM_2.5_ suspension concentration through pre-experiments is detailed in Session S1 of the Supplementary Information. The results are shown in Figure S1.

### 2.4. Cell Culture

Human lung epithelial cells, A549 (Shanghai Fuheng Biotechnology, China), were cultured in F12K medium (Shanghai Fuheng Biotechnology, China) supplemented with 10% fetal bovine serum (FBS, Suzhou Ekosai, Suzhou, China) at 37 °C in an atmosphere of 5% CO_2_. When the cells reached approximately 80% confluence, they were digested with trypsin and used for plating or passaging.

### 2.5. Cytotoxicity Assay

Cell viability was determined by CCK8 assay (cell suspension concentration was 1 × 10^5^ cells/mL). Each well had a volume of 100 μL. After 24 h of incubation, 240 mg/L of the staining masterbatch was added to the 96-well plates at a volume of 100 μL per well. Three replicate wells were established for each concentration, along with a blank group and a control group. Following an additional 24 h of incubation, 10 μL of CCK-8 was added to each well, and the plate was incubated in the dark for 1 h. Finally, the optical density (OD) of the cell suspensions was measured using an enzyme-labeled instrument (Shanghai Kewa Bio-engineering, Shanghai, China) at a wavelength of 450 nm.

Glutathione peroxidase (GSH-Px), ROS, superoxide dismutase (SOD), LDH, TNF-α, and interleukin-6 (IL-6) in the supernatant were measured by enzyme-linked immunosorbent assay (ELISA) kits (Jiangsu Sumeike Biological Technology, Dongtai, China). Cells were inoculated into 96-well plates following the aforementioned method. After 24 h of incubation, the medium was aspirated, PM_2.5_ staining solution was added, and incubation continued for an additional 24 h. The supernatant was aspirated and transferred to the kits for analysis, and the OD of each well was measured at a wavelength of 450 nm. Among them, the ELISA determination results of the blank filter are shown in Figure S2 of the Supplementary Information.

### 2.6. UPLC-MS-Based Untargeted Metabolomics Analysis

A549 cells were inoculated into six-well plates, with each well containing 2 mL of medium. The cells were incubated in a 5% CO_2_ incubator at 37 °C until they reached a density of 1 × 10^6^ cells. Then, 500 μL of staining solution was added to the medium. The cells were further incubated for 24 h. After incubation, the medium was aspirated, and the cells were washed twice with 2 mL of cold PBS. Subsequently, the cells were gently scraped on ice using 1 mL of PBS, collected, and centrifuged at 800× *g* for 5 min. The supernatant was discarded, and 4 mL of pre-cooled (−80 °C) 80% (*v*/*v*) HPLC-grade methanol was added to precipitate the cells. The mixture was vortexed for 1 min and then incubated for 30 min at −80 °C. The samples were centrifuged for 10 min at 4 °C and 4000× *g*. The collected supernatant was dried using a SpeedVac concentrator. The dried metabolite samples were stored at −80 °C until further analysis by mass spectrometry. To evaluate the stability and reproducibility of the analytical method, quality control (QC) samples were prepared from aliquots of each sample.

The metabolites produced following exposure of A549 cells to PM_2.5_ were analyzed using UPLC-MS equipped with an HSS T3 column (2.1 × 100 mm, 1.8 μm). The UPLC-MS system included liquid chromatography (UPLC, Waters ACQUITY UPLC I-Class, Milford, MA, USA) and mass spectrometry (Thermo Fisher Q Exactive). Ionization was achieved using positive (ESI^+^) and negative (ESI^−^) electrospray ionization (ESI) sources. The HSS T3 column was operated at a flow rate of 0.4 mL/min with an injection volume of 3 μL. The mobile phase comprised H_2_O (A) and acetonitrile (ACN, B), both containing 0.1% formic acid. The mass scan range was set to 100–1500 amu. Instrument calibration was conducted prior to sample introduction.

### 2.7. Statistical Analysis

The results were analyzed using one-way ANOVA (ANOVA, GraphPad Prism 10 software). The *p*-value < 0.05 indicated statistical significance, while a *p*-value < 0.01 indicated high significance. All statistical metabolomics analyses were performed using MetaboAnalyst v5.0 online platform (https://www.metaboanalyst.ca accessed on 10 June 2025). Partial least squares discriminant analyses (PLS-DAs) were utilized to model the relationship between metabolite levels and sample subgroups, with colored ellipses indicating the 95% confidence level coverage. Venn diagrams were created using the EVenn website (Available online: http://www.ehbio.com/test/venn/ (accessed on 10 June 2025)). Differential metabolites were deemed statistically significant when *p* < 0.1 and fold change (FC) > 2. Enrichment analyses were carried out using the Small Molecule Pathway Database (SMPDB).

## 3. Results

### 3.1. Characterization of PM_2.5_ Chemical Components in the Plateau City

#### 3.1.1. PM_2.5_ Mass Concentration

**Figure 2:**
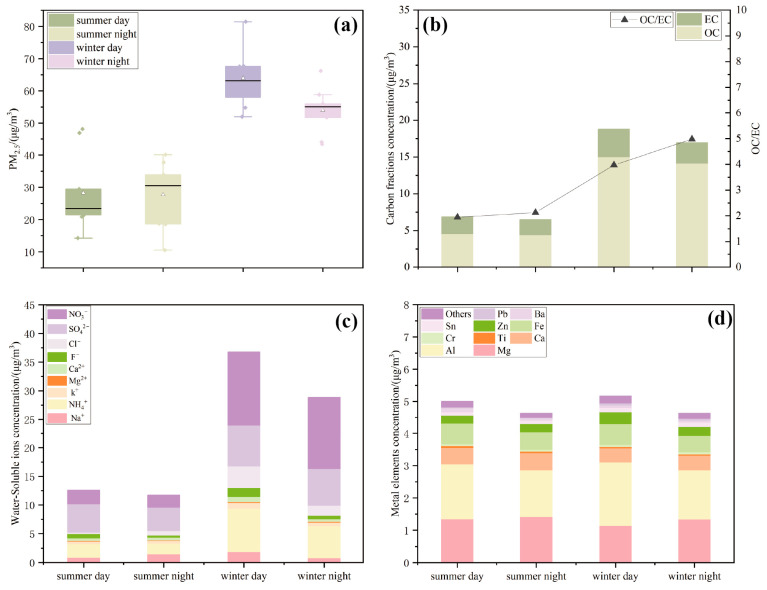
Changes of PM_2.5_ mass and component concentrations in summer and winter: (**a**) mass concentration; (**b**) carbon fractions; (**c**) water-soluble ions; (**d**) metallic elements. (White triangular symbols represent the mean values, and the black solid lines represent the median values).

Figure 2a displays the PM_2.5_ mass concentration in Xining during the summer and winter sampling periods. The average PM_2.5_ mass concentration in Xining was 42.87 ± 18.12 μg/m^3^. The distribution of PM_2.5_ mass concentration exhibited distinct seasonal characteristics, being significantly higher in winter (58.63 ± 9.02 μg/m^3^) than in summer (27.98 ± 10.26 μg/m^3^), by approximately 2.10 times. Additionally, the PM_2.5_ mass concentrations presented diurnal variations, with daytime levels mostly surpassing nighttime levels. Specifically, the daytime concentrations were 1.02 times higher than those of nighttime in summer and 1.18 times higher in winter.

#### 3.1.2. Concentration of Carbonaceous Components in PM_2.5_ Samples

Figure 2b illustrates the variations in mass concentrations and ratios of OC and EC in PM_2.5_ during summer and winter. OC predominated in all PM_2.5_ samples in Xining. During the sampling period, the average mass concentrations of OC in summer were 4.48 ± 2.87 μg/m^3^ and 2.21 ± 1.60 μg/m^3^ for EC, whereas in winter, the average concentrations were 14.54 ± 5.74 μg/m^3^ for OC and 3.28 ± 1.77 μg/m^3^ for EC. The variations in the carbonaceous components were evident in different seasons. During both seasons, the mass concentrations of OC and EC were higher during the day than during the night. Furthermore, the OC/EC ratios were higher in winter than in summer. Notably, nighttime OC/EC ratios exceeded daytime ratios in both seasons, with values of 4.98 in winter and 2.13 in summer, both exceeding 2 and indicating the production of secondary organic carbon (SOC).

#### 3.1.3. Concentration of Water-Soluble Ions in PM_2.5_ Samples

Figure 2c indicates that the water-soluble ion concentrations were significantly higher in winter than in summer and higher during the day than at night. In summer, the water-soluble ion concentrations, listed from highest to lowest, were SO_4_^2−^ > NO_3_^−^ > NH_4_^+^ > Na^+^ > F^−^ > Cl^−^ > K^+^ > Ca^2+^ > Mg^2+^. In winter, the order was NO_3_^−^ > SO_4_^2−^ > NH_4_^+^ > Cl^−^ > Na^+^ > F^−^ > K^+^ > Ca^2+^ > Mg^2+^. SO_4_^2−^, NO_3_^−^, and NH_4_^+^ (collectively termed as SNA) were the principal water-soluble ions in PM_2.5_ samples, with winter SNA constituting 79.27% of the total winter ions and summer SNA accounting for 73.21% of the total. This suggests that nitrate, sulfate, and ammonium salts were predominant contributors to PM_2.5_ water-soluble ions in Xining.

#### 3.1.4. Concentration of Metallic Elements in PM_2.5_ Samples

Figure 2d depicts the metallic elements across the seasons and times of day. The top ten metals were selected for plotting as they represented 80% of the total metal content, while other elements, which accounted for a smaller proportion, were combined for the analysis. The average mass concentrations of metallic elements exhibited pronounced seasonal and diurnal variations, being higher in winter than in summer and during the day compared to night; however, the differences were relatively minor. Overall, the mass concentrations of Mg, Al, Fe, and Ca were elevated in both summer and winter, constituting the primary metallic elements in PM_2.5_ in Xining. In contrast, Bi, Sb, Mo, and Se were present at lower levels, yet their toxicity should not be overlooked.

### 3.2. In Vitro Cytotoxicity of PM_2.5_ in the Plateau City

#### 3.2.1. Effects of PM_2.5_ on A549 Cell Viability

**Figure 3:**
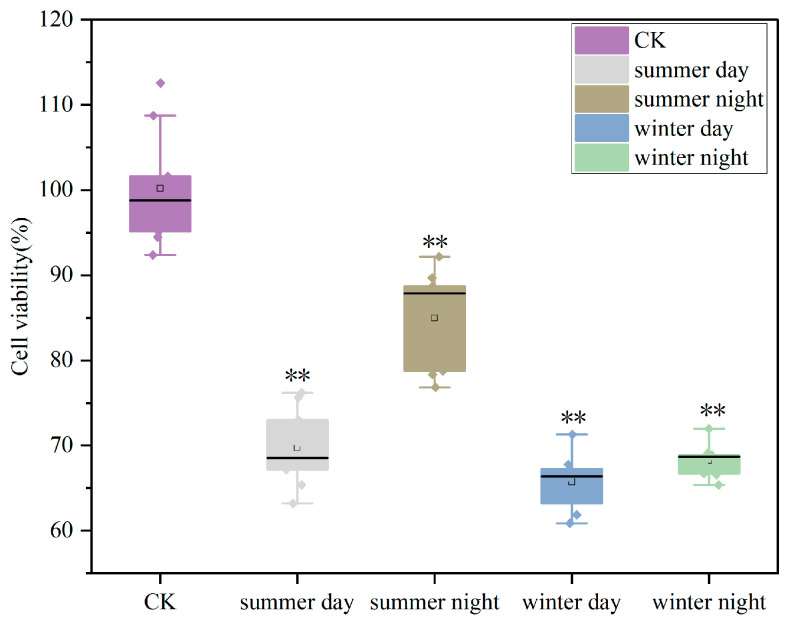
Effects of daytime and nighttime PM_2.5_ exposure on A549 cell survival rates during summer and winter seasons. CK: control check. Compared with CK, ** is *p* < 0.01.

As shown in Figure 3, the cell survival rates significantly decreased after 24 h of exposure to PM_2.5_ compared to the blank control group. This effect was more pronounced in winter, with an average survival rate of approximately 67.06%. The survival rates were lower in the daytime than at nighttime, with a reduction of 30.45% in summer and 34.46% in winter compared to the control group. Therefore, the cell toxicities were generally higher during the daytime.

#### 3.2.2. Oxidative Stress of A549 Cells Induced by PM_2.5_ Exposure

**Figure 4:**
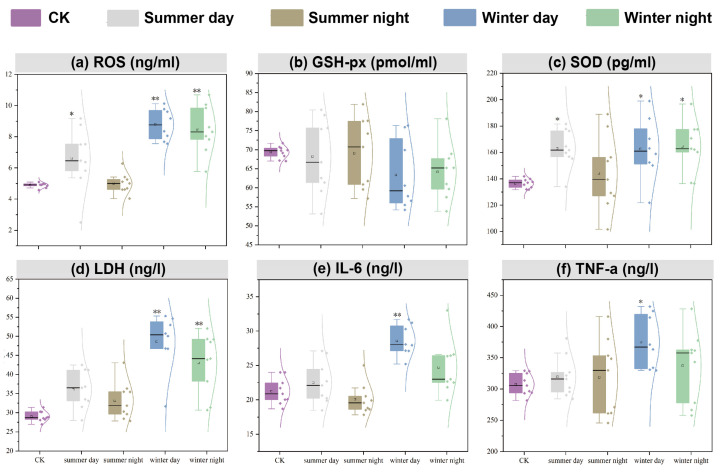
Effects of PM_2.5_ on oxidative stress, membrane injury, and inflammatory damage in A549 cells: (**a**–**c**) oxidative stress; (**d**) cell membrane damage; (**e**,**f**) inflammatory injury. CK: control check. Compared with CK, * is *p* < 0.05; ** is *p* < 0.01. (Curves of different colors represent the normal distribution curves of different groups.)

Figure 4a–c illustrates the levels of ROS, GSH-px, and SOD in the supernatant of A549 cells following 24 h of exposure to PM_2.5_. Both ROS and SOD production were elevated in all samples, except during summer nights, when the increase was not significant. Specifically, ROS production in winter reached 8.62 ng/mL, representing 1.77 times that of the control group, while SOD production was 163.64 pg/mL, equivalent to 1.20 times that of the control group. In contrast to other oxidative stress indicators, GSH-px production was low in winter and remained relatively low during the daytime. Overall, these results suggest that stronger oxidative stress responses were induced in winter and during the daytime across both seasons.

#### 3.2.3. Cell Membrane Injury of A549 Cells Induced by PM_2.5_ Exposure

Figure 4d illustrates the effect of PM_2.5_ on cell membrane injury. All PM_2.5_ samples collected in Xining induced LDH production. Consistent with the oxidative stress indicators, LDH production was higher in winter than in summer, and greater during the daytime compared to nighttime. Specifically, LDH production in winter was measured at 45.65 ng/L, which was 1.58 times higher than that of the control group and 1.32 times higher than in summer, indicating that PM_2.5_ had the most significant impact on cell membrane damage during winter. During the daytime, LDH production was 1.11 times greater than that at nighttime.

#### 3.2.4. Inflammatory Injury of A549 Cells Induced by PM_2.5_ Exposure

As shown in Figure 4e–f, both IL-6 and TNF-a production were higher in winter than in summer and greater during the daytime compared to nighttime. IL-6 and TNF-a production were induced by PM_2.5_ exposure, except during summer nights. The productions at daytime were notably higher in winter, with IL-6 levels being 1.34 times and TNF-a levels 1.22 times greater than the control groups. Additionally, the strongest inflammatory injury occurred during winter daytime.

#### 3.2.5. Correlation Between the Cytotoxicity and Chemical Components of PM_2.5_

**Figure 5:**
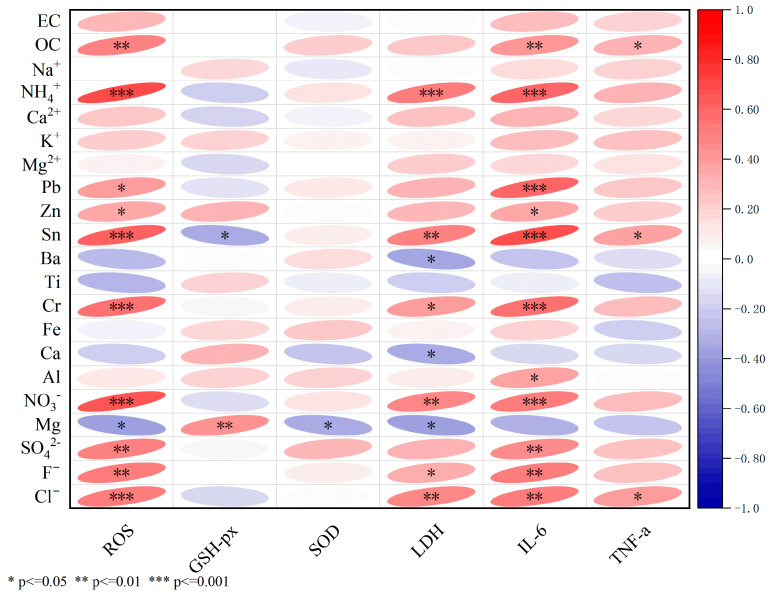
Correlation analysis of PM_2.5_ chemical components with toxicological indexes. (The tilt direction of ellipses in different colors indicates the positive or negative nature of the correlation: ellipses tilt to the left for a positive correlation and to the right for a negative correlation).

Figure 5 illustrates the correlations between cytotoxicity and the chemical components of PM_2.5_ samples, including carbonaceous components, water-soluble ions, and metallic elements. Most chemical components in the PM_2.5_ samples exhibited a negative correlation with GSH-px, suggesting that the initial levels of GSH-px were depleted in the organism, with a particularly significant negative correlation observed with Sn. Conversely, these components showed significantly positive correlations with ROS, LDH, IL-6, and TNF-a, suggesting their potential contribution to oxidative stress, cell membrane damage, and inflammatory responses in Xining. Compared to other toxicological indicators, ROS and IL-6 exhibited the strongest responses to the chemical components of PM_2.5_. OC, Cl^−^, F^−^, Sn, Cr, SO_4_^2−^, Pb, Zn, Mg, NO_3_^−^, and NH_4_^+^ might demonstrate greater cytotoxicity than other components.

### 3.3. Metabolite Effects of PM_2.5_ Exposure on A549 Cells in the Plateau City

Significant differences in metabolic profiles were observed (see Figure S3) between the sample and control groups through the partial least squares discriminant analysis (PLS-DA). It suggests that PM_2.5_ samples in Xining induced substantial metabolic perturbations in A549 cells.

**Figure 6:**
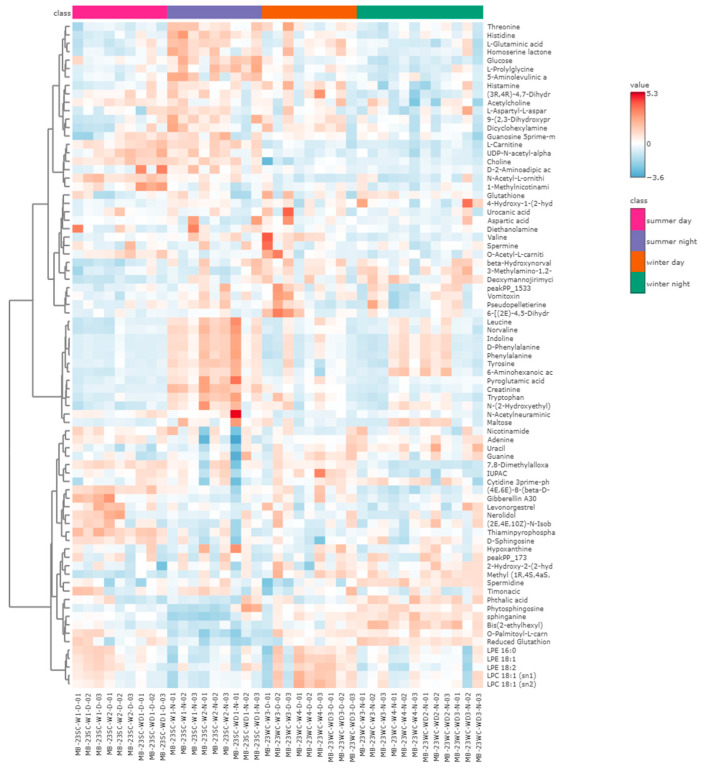
Heat map of metabolite abundances between samples.

We subsequently performed a comprehensive comparison of metabolite expression levels among the four sample groups. Figure 6 indicates the abundances of specific metabolites across the groups. Notably, significant differences in metabolite abundance were observed between the summer daytime and nighttime groups, whereas the variances between the winter daytime and nighttime groups were comparatively minor.

**Figure 7:**
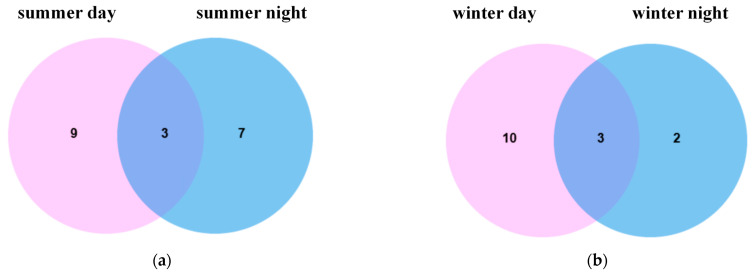

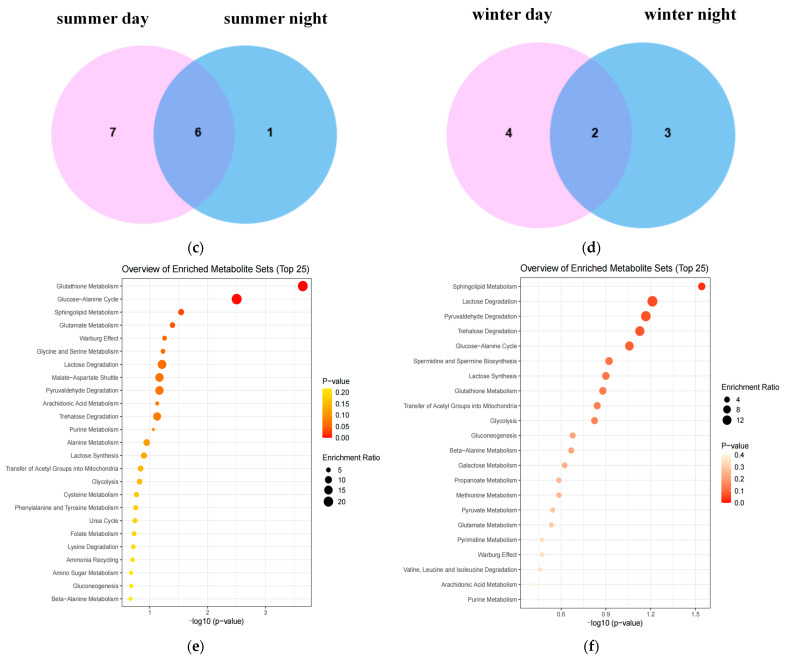
(**a**,**b**) Venn diagram of upregulation of metabolites in A549 cells incubated with summer and winter samples; (**c**,**d**) Venn diagram of downregulation of metabolites in A549 cells incubated with summer and winter samples; (**e**,**f**) map of differential cellular metabolic pathways after exposure to PM_2.5_ samples in summer and winter.

Compared to the controls, there were three and six metabolites shared between daytime and nighttime upregulation and downregulation, respectively, in summer, and three and two metabolites in winter, with levels significantly higher in summer than in winter, as shown in Figure 7a–d. Further analysis of the biological pathways associated with the summer differential metabolites highlighted significant effects predominantly on glutathione metabolism, the glucose-alanine cycle, sphingolipid metabolism, and glutamate metabolism. Differential metabolites in winter primarily affected sphingolipid metabolism, as seen in Figure 7e,f.

**Figure 8:**
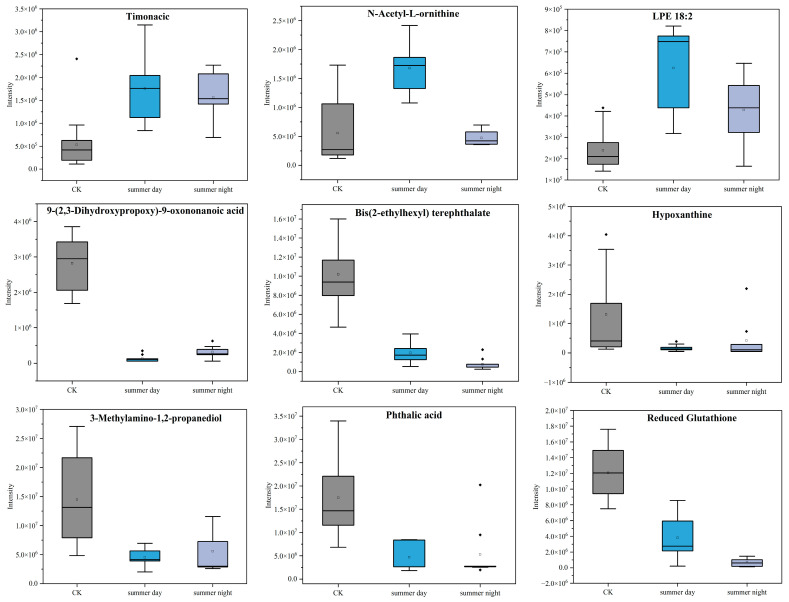
Changes in peak intensity of shared metabolite peaks during summer, day and night. (Square symbols represent the mean values, and black solid lines represent the median lines).

**Figure 9:**
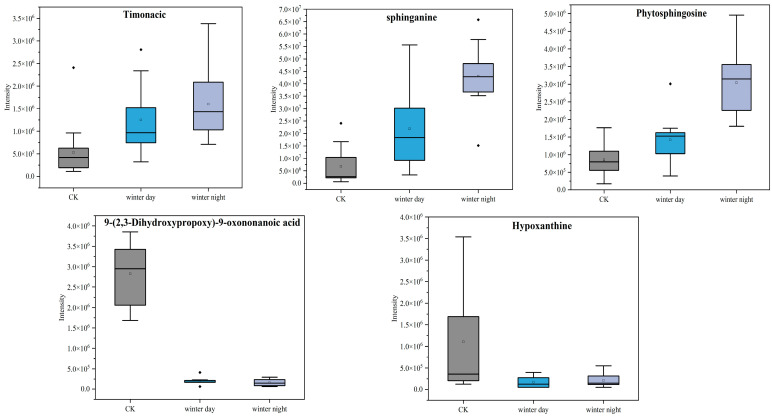
Changes in peak intensity of shared metabolite peaks during winter, day and night. (Square symbols represent the mean values, and black solid lines represent the median lines).

Figure 8 shows the peak intensities of metabolites in summer. It reveals that nine metabolites were co-produced diurnally during summer, including timonacic, N-Acetyl-L-ornithine, LysoPE (18:2), 9-(2,3-Dihydroxypropoxy)-9-oxononanoic acid, bis (2-ethylhexyl) terephthalate, hypoxanthine, 3-Methylamino-1,2-propanediol, phthalic acid, and reduced glutathione. The peak intensities of three upregulated metabolites—timonacic, N-Acetyl-L-ornithine, and LysoPE (18:2)—were higher during the day than at night, while the four downregulated metabolites—9-(2,3-Dihydroxypropoxy)-9-oxononanoic acid, hypoxanthine, 3-Methylamino-1,2-propanediol, and phthalic acid—were lower during the day. In winter, five metabolites were identified during both day and night (Figure 9), including timonacic, sphinganine, phytosphingosine, 9-(2,3-Dihydroxypropoxy)-9-oxononanoic acid, and hypoxanthine. Among these, hypoxanthine exhibited the same peak intensity pattern as observed in summer, while the others displayed the opposite pattern. Timonacic, 9-(2,3-Dihydroxypropoxy)-9-oxononanoic acid, and hypoxanthine were metabolites common in both summer and winter.

## 4. Discussion

The Qinghai-Tibet Plateau is a sensitive and fragile zone in response to climatic and ecological changes, serving as a critical indicator of global environmental dynamics [20]. Along with the increase of human activities in the plateau, fossil-fuel-related emission sources have been the main contributors of PM_2.5_ in Xining [25]. The cytotoxicity properties and mechanisms of PM_2.5_ exposure in this high-altitude city remain understudied, which has critical implications for public health protection and air quality management.

In this study, the average PM_2.5_ mass concentration in Xining was lower than the limit value of the Chinese National Ambient Air Quality Standard (GB 3095-2012) [26] in China, which was set at 75 μg/m^3^. It was also obviously lower than those observed in the plain cities such as Nanjing [27], Zibo [28], Beijing [29], and Wuhan [30]. Differences in atmospheric PM_2.5_ concentrations between regions may be attributed to factors such as topography, meteorological conditions, and economic development levels. Elevated PM_2.5_ concentrations in Xining during winter might result from emissions originating from anthropocentric sources, particularly domestic coal combustion [25].

The chemical analysis of PM_2.5_ components provides a basis for analyzing aerosol sources and assessing health risks [25]. The mass concentrations of OC in Xining significantly exceeded those of EC. The presence of secondary organic carbon is indicated when the OC/EC ratio in ambient aerosol exceeds 2 [31]. The OC/EC ratio exceeded 2 for all samples except during daytime in summer (Figure 2b), indicating the prevalence of SOC in PM_2.5_ in Xining. Elevated OC/EC ratios at night in both seasons suggested increased formation of secondary organic aerosols, consistent with findings reported in Xi’an [32,33]. Furthermore, this study found that the OC/EC ratio in Xining was lower than in other highland regions, including Qinghai Lake (elevation 3200 m) [34], Mount Everest (elevation 4276 m) [35], and Namucuo (elevation 4730 m) [36], with the highest ratio observed in Namucuo (up to 17.6). Strong photochemical reactions, driven by extensive solar radiation, were likely the primary factor contributing to the elevated OC/EC ratio [37]. SNAs were the predominant water-soluble ions in PM_2.5_ during both summer and winter (Figure 2c), typically formed through the chemical transformation of gaseous precursors, including SO_2_, NO_x_, and NH_3_, directly emitted into the atmosphere [38]. Atmospheric SNAs were readily adsorbed onto the surfaces of fine particles and intermixed with organic matter [39]. A high mass fraction of inorganic ions (SNA) at the sampling sites indicated the production of secondary inorganic pollution in Xining. The PM_2.5_ source apportionment in Xining indicated a growing contribution from transportation sources, where emissions of NO_x_ were converted into NO_3_^−^, potentially resulting in increased NO_3_^−^ concentrations. Additionally, the proportion of secondary sulfate had been observed to rise annually, leading to an increase in SO_4_^2−^ levels [25]. Toxicological studies indicated that an increase in the proportions of sulfate and nitrate in PM_2.5_ were associated with a relative increase in mortality rates [40]. The total concentration of water-soluble ions in Xining was 22.09 μg/m^3^, which was lower than that observed in plain cities such as Wuhan [41] and Dongying [42]. Although the proportion of metallic elements in PM_2.5_ was low, their significant harmful effects on human health should not be overlooked [43]. Winter meteorological conditions hindered pollutant dispersion, resulting in elevated levels of metallic elements, consistent with the results of PM_2.5_ measurements in the areas with varying traffic densities [44]. The elevated concentration of Al among the measured elements was attributed to the significant presence of crustal elements brought in by soil dust. Additionally, the growing contribution of transportation sources in Xining contributed to increased levels of elements such as Fe and Mg [25]. Fe primarily originated from road dust produced by vehicle traffic. Mg and its alloys were the important materials for automobiles, which increased the risk of Mg emissions [45].

In vitro cytotoxicity tests for PM_2.5_ exposure have been extensively applied to clarify its toxicological effects [46]. The risks associated with PM_2.5_ exposure were confirmed by a significant decrease in cell viability, as measured by CCK8 after 24 h of PM_2.5_ exposure in all samples (Figure 3). This decrease was more pronounced in winter, a seasonal trend consistent with results obtained from urban samples [47]. PM_2.5_ exposure initially stimulated oxidative stress in the cells [48]. The release of ROS indicated the onset of oxidative stress (Figure 4a). Secretion levels of SOD and ROS were comparable (Figure 4c), with heightened oxidative stress observed in winter and during daytime in both seasons. GSH-Px, an important metabolic regulator in cells, activated various enzymes and bound to peroxides and free radicals, thereby mitigating the damaging effects of these radicals [49]. In this study, GSH-Px production was lower in winter and during daytime across both seasons, confirming its role in reducing cellular antioxidant capacity [50]. IL-6, TNF-α, and LDH were indicative of inflammatory reactions and membrane injury for A549 cells. In this study, the mean production levels of ROS, IL-6, SOD, LDH, and TNF-α were higher than those of the control group by factors of 1.77, 1.13, 1.16, 1.39, and 1.10, respectively. Conversely, GSH-Px decreased to 0.95 times that of the control group levels. The response degrees of ROS, TNF-α, IL-6, and SOD in our study were higher than those observed in Nanjing, whereas the levels of LDH and GSH-Px were lower than those in Nanjing [8]. This illustrated that the discrepancy in PM_2.5_ components across different cities resulted in variations in toxic indices. Correlation results in Figure 5 indicate that ROS and IL-6 exhibited the strongest correlation to most of PM_2.5_ chemical fractions, including OC, Cl^−^, F^−^, Sn, Cr, SO_4_^2−^, Pb, Zn, Mg, NO_3_^−^, and NH_4_^+^. A consistent conclusion was presented in Canada, revealing a significant correlation between the concentrations of metallic elements, including Mg and Zn, in PM_2.5_ and A549 cell viability and ROS production [51]. Furthermore, this study identified a strong positive correlation between NH_4_^+^, SO_4_^2−^, NO_3_^−^, and IL-6 production. NH_4_^+^, SO_4_^2−^, and NO_3_^−^ are linked to oxidative stress and inflammatory responses, with sulfate potentially causing endothelial dysfunction [50].

The specific metabolic pathways related to PM_2.5_ exposure in Xining were subsequently explored in depth through a UPLC-MS-based metabolomics approach (Figure 7). Sphingolipid metabolism, a subset of lipid metabolism, was particularly affected by exposure of A549 cells to PM_2.5_ in both summer and winter. It has been reported that long-term exposure to PM_2.5_ would lead to alterations in sphingolipid metabolism [52]. Sphingolipids were key components of cell membranes, comprising approximately 10% to 20% of total membrane lipids. Moreover, sphingolipid metabolites have been shown to be closely associated with the inflammatory response in alveolar type II cells [53]. During the inflammatory response, innate and adaptive immune cells migrated to the site of infection or injury, activating the cytokine network to protect the host. Meanwhile, sphingolipid metabolites played a crucial role in the transport and function of these immune cells [54]. Glutathione was an important antagonist of oxidative stress that was part of amino acid metabolism [55]. Disruption of this pathway can impair the body’s ability to combat oxidative stress. Previous studies indicate that glutathione, functioning as a free radical scavenger in vivo, not only inhibited the formation of free radicals through enzyme catalysis but also directly reacted with free radicals to convert them into stabilizing molecules [56]. Furthermore, the main metabolic pathway in summer was acid metabolism. Amino acids were essential for cell growth and reproduction. They served as precursors for protein synthesis and were integral to nucleotide and lipid biosynthesis, as well as being important sources of energy metabolism and precursors for various metabolites [57]. The amino acids and their metabolites examined in our results participated in various reactions, including immune responses, cell signaling, and hormone formation [58]. Thus, glutathione metabolism, amino acid metabolism, and sphingolipid metabolism were crucial pathways influencing the induction of oxidative stress and inflammatory responses in cells exposed to PM_2.5_ in Xining. In traffic-related environmental samples, short-term exposure to PM_2.5_ also caused significant disturbances in amino acid and lipid metabolism. These two pathways may be associated with increased contributions from traffic sources, which was consistent with the results of source apportionment in Xining [59]. Different metabolic pathways exposed to PM_2.5_ have been implicated in some low-altitude cities. For instance, glycerophospholipid, purine, and sphingolipid metabolism were the most critical pathways in Tangshan (altitude 27.8 m) [60]. Among them, sphingolipid metabolism was also found in Xining. In Xiamen (altitude 63 m), PM_2.5_ disrupted three key metabolic pathways in A549 cells, including the cit-rate cycle, amino acid biosynthesis, and glutathione metabolism [19]. Thus, metabolic pathways may differ across cities due to variations in meteorological conditions, altitude, emission source contributions, and other factors.

The peak intensities of common metabolites both in summer and winter revealed that sphingomyelin was significantly upregulated in winter. Following exposure of A549 cells to PM_2.5_, sphingomyelin levels were found to be significantly elevated at the metabolic level. The likely mechanism was that PM_2.5_ activated the protein kinase B (PKB) signaling pathway in vivo, resulting in increased spingomyelin levels [61]. In summer, LysoPE (18:2) levels were improved, and akin to platelets, lysophospholipids played a pivotal role in blood coagulation [62]. In our results, exposure to increased PM_2.5_ concentrations gave rise to elevated production of lysophospholipids, which was consistent with findings observed in mice following in vivo exposure to PM_2.5_ [59]. Timonacic, 9-(2,3-dihydroxypropoxy)-9-oxononanoic acid, and hypoxanthine were common metabolites detected in both seasons. Notably, hypoxanthine belonged to the purine category, and its presence led to the signal transduction of purinergic receptors, which was linked to lung injury [63].

## 5. Conclusions

To understand the human health impacts of exposure to PM_2.5_ in plateau cities, this study analyzed the seasonal and diurnal variations of PM_2.5_ chemical components and cytotoxicity, further elucidating the toxicity mechanisms using metabolomics. The mass concentrations of PM_2.5_ in Xining were 27.98 ± 10.26 μg/m^3^ in summer and 58.63 ± 9.02 μg/m^3^ in winter, representing a 2.1-fold increase compared to summer levels. OC, SO_4_^2−^, NO_3_^−^, and NH_4_^+^ were identified as the primary chemical components of PM_2.5_, while Mg, Al, Fe, and Ca represented a significant proportion of the metallic elements. Toxicological studies indicated that the toxicity indicator most significantly affected by PM_2.5_ exposure in summer was LDH, while in winter, the most pronounced indicator was ROS. Key components inducing oxidative stress and inflammatory responses in cells included OC, Cl^−^, F^−^, Sn, Cr, SO_4_^2−^, Pb, Zn, Mg, NO_3_^−^, and NH_4_^+^. Metabolomic analyses indicated that glutathione metabolism, amino acid metabolism, and sphingolipid metabolism were critical pathways influencing toxic responses in cells. Notably, timonacic, 9-(2,3-dihydroxypropoxy)-9-oxononanoic acid, and hypoxanthine were identified as common metabolites in both seasons.

## Data Availability

The data presented in this study are available upon request from the corresponding author.

## Supplementary Materials

The following supporting information can be downloaded at: https://www.mdpi.com/article/10.3390/toxics13090729/s1, Session S1. Pre-experiments for the concentrations of PM_2.5_ suspensions; Figure S1: Changes in cell viability at different concentrations; Figure S2: Results of in vitro cytotoxicity for blank experiments; Figure S3: PLS-DA scatterplot: (a) summer day; (b) summer night; (c) winter day; (d) winter night.

## References

[1] Zhou L., Chen X., Tian X. (2018). The Impact of Fine Particulate Matter (PM_2.5_) on China’s Agricultural Production from 2001 to 2010. J. Clean. Prod..

[2] Chen H.-S., Lin Y.-C., Chiueh P.-T. (2023). Nexus of Ecosystem Service-Human Health-Natural Resources: The Nature-Based Solutions for Urban PM_2.5_ Pollution. Sustain. Cities Soc..

[3] Cohen A.J., Brauer M., Burnett R., Anderson H.R., Frostad J., Estep K., Balakrishnan K., Brunekreef B., Dandona L., Dandona R. (2017). Estimates and 25-Year Trends of the Global Burden of Disease Attributable to Ambient Air Pollution: An Analysis of Data from the Global Burden of Diseases Study 2015. Lancet.

[4] Xie M., Chen X., Hays M.D., Holder A.L. (2019). Composition and Light Absorption of N-Containing Aromatic Compounds in Organic Aerosols from Laboratory Biomass Burning. Atmos. Chem. Phys..

[5] Xiong T.-T., Leveque T., Austruy A., Goix S., Schreck E., Dappe V., Sobanska S., Foucault Y., Dumat C. (2014). Foliar Uptake and Metal(Loid) Bioaccessibility in Vegetables Exposed to Particulate Matter. Environ. Geochem. Health.

[6] Jahanzaib M., Iqbal S., Shoukat S., Park D. (2025). Personal Exposure Assessment of Respirable Particulate Matter Among University Students Across Microenvironments During the Winter Season Using Portable Monitoring Devices. Toxics.

[7] Pope C.A., Dockery D.W. (2006). Health Effects of Fine Particulate Air Pollution: Lines That Connect. J. Air Waste Manag. Assoc..

[8] Knaapen A.M., Borm P.J.A., Albrecht C., Schins R.P.F. (2004). Inhaled Particles and Lung Cancer. Part A: Mechanisms. Int. J. Cancer.

[9] Chen Q., Luo X.-S., Chen Y., Zhao Z., Hong Y., Pang Y., Huang W., Wang Y., Jin L. (2019). Seasonally Varied Cytotoxicity of Organic Components in PM_2.5_ from Urban and Industrial Areas of a Chinese Megacity. Chemosphere.

[10] Lippmann M. (2014). Toxicological and Epidemiological Studies of Cardiovascular Effects of Ambient Air Fine Particulate Matter (PM_2.5_) and Its Chemical Components: Coherence and Public Health Implications. Crit. Rev. Toxicol..

[11] Dieme D., Cabral-Ndior M., Garçon G., Verdin A., Billet S., Cazier F., Courcot D., Diouf A., Shirali P. (2012). Relationship between Physicochemical Characterization and Toxicity of Fine Particulate Matter (PM_2.5_) Collected in Dakar City (Senegal). Environ. Res..

[12] Perrone M.G., Gualtieri M., Ferrero L., Porto C.L., Udisti R., Bolzacchini E., Camatini M. (2010). Seasonal Variations in Chemical Composition and in Vitro Biological Effects of Fine PM from Milan. Chemosphere.

[13] Pang Y., Huang W., Luo X.-S., Chen Q., Zhao Z., Tang M., Hong Y., Chen J., Li H. (2020). In-Vitro Human Lung Cell Injuries Induced by Urban PM_2.5_ during a Severe Air Pollution Episode: Variations Associated with Particle Components. Ecotoxic. Environ. Safety.

[14] Zhou Q., Chen J., Zhang J., Zhou F., Zhao J., Wei X., Zheng K., Wu J., Li B., Pan B. (2022). Toxicity and Endocrine-Disrupting Potential of PM_2.5_: Association with Particulate Polycyclic Aromatic Hydrocarbons, Phthalate Esters, and Heavy Metals. Environ. Pollut..

[15] Wang D., Pakbin P., Shafer M.M., Antkiewicz D., Schauer J.J., Sioutas C. (2013). Macrophage Reactive Oxygen Species Activity of Water-Soluble and Water-Insoluble Fractions of Ambient Coarse, PM_2.5_ and Ultrafine Particulate Matter (PM) in Los Angeles. Atmos. Environ..

[16] Nobels I., Vanparys C., Van den Heuvel R., Vercauteren J., Blust R. (2012). Added Value of Stress Related Gene Inductions in HepG2 Cells as Effect Measurement in Monitoring of Air Pollution. Atmos. Environ..

[17] Happo M., Markkanen A., Markkanen P., Jalava P., Kuuspalo K., Leskinen A., Sippula O., Lehtinen K., Jokiniemi J., Hirvonen M.-R. (2013). Seasonal Variation in the Toxicological Properties of Size-Segregated Indoor and Outdoor Air Particulate Matter. Toxicol. Vitr..

[18] Becker S., Dailey L.A., Soukup J.M., Grambow S.C., Devlin R.B., Huang Y.-C.T. (2005). Seasonal Variations in Air Pollution Particle-Induced Inflammatory Mediator Release and Oxidative Stress. Environ. Health Perspect..

[19] Huang Q., Zhang J., Luo L., Wang X., Wang X., Alamdar A., Peng S., Liu L., Tian M., Shen H. (2015). Metabolomics Reveals Disturbed Metabolic Pathways in Human Lung Epithelial Cells Exposed to Airborne Fine Particulate Matter. Toxicol. Res..

[20] Zhang Y., Li Y., Shi Z., Wu J., Yang X., Feng L., Ren L., Duan J., Sun Z. (2018). Metabolic Impact Induced by Total, Water Soluble and Insoluble Components of PM_2.5_ Acute Exposure in Mice. Chemosphere.

[21] Hu Z., Kang S., Li C., Yan F., Chen P., Gao S., Wang Z., Zhang Y., Sillanpää M. (2017). Light Absorption of Biomass Burning and Vehicle Emission-Sourced Carbonaceous Aerosols of the Tibetan Plateau. Environ. Sci. Pollut. Res. Int..

[22] Zheng H., Wu D., Wang S., Li X., Jin L.N., Zhao B., Li S., Sun Y., Dong Z., Wu Q. (2025). Control of Toxicity of Fine Particulate Matter Emissions in China. Nature.

[23] Xing Z., Yang T., Shi S., Meng X., Chen R., Long H., Hu Y., Chai D., Liu W., Tong Y. (2023). Ambient Particulate Matter Associates with Asthma in High Altitude Region: A Population-Based Study. World Allergy Organ. J..

[24] Zhou L., Wang Y., Wang Q., Ding Z., Jin H., Zhang T., Zhu B. (2023). The Interactive Effects of Extreme Temperatures and PM_2.5_ Pollution on Mortalities in Jiangsu Province, China. Sci. Rep..

[25] Zhang G., Zhao X., Liu Y., Zheng Z., Chen Q., Geng C., Wang X., Han B., Bai Z. (2024). Comparative Source Apportionment of PM_2.5_ for 2014/2019 at a Plateau City: Implications for Air Quality Improvement in High-Altitude Areas. Atmos. Pollut. Res..

[26] (2012). Ambient Air Quality Standards.

[27] Li H., Zhao Z., Luo X.-S., Fang G., Zhang D., Pang Y., Huang W., Mehmood T., Tang M. (2022). Insight into Urban PM_2.5_ Chemical Composition and Environmentally Persistent Free Radicals Attributed Human Lung Epithelial Cytotoxicity. Ecotoxicol. Environ. Saf..

[28] Zhang J., Zhou X., Wang Z., Yang L., Wang J., Wang W. (2018). Trace Elements in PM_2.5_ in Shandong Province: Source Identification and Health Risk Assessment. Sci. Total Environ..

[29] Batterman S., Xu L., Chen F., Chen F., Zhong X. (2016). Characteristics of PM_2.5_ Concentrations across Beijing during 2013–2015. Atmos. Environ..

[30] Zhang F., Wang Z., Cheng H., Lv X., Gong W., Wang X., Zhang G. (2015). Seasonal Variations and Chemical Characteristics of PM_2.5_ in Wuhan, Central China. Sci. Total Environ..

[31] Hao Y., Gao C., Deng S., Yuan M., Song W., Lu Z., Qiu Z. (2019). Chemical Characterisation of PM_2.5_ Emitted from Motor Vehicles Powered by Diesel, Gasoline, Natural Gas and Methanol Fuel. Sci. Total Environ..

[32] Bozlaker A., Peccia J., Chellam S. (2017). Indoor/Outdoor Relationships and Anthropogenic Elemental Signatures in Airborne PM_2.5_ at a High School: Impacts of Petroleum Refining Emissions on Lanthanoid Enrichment. Environ. Sci. Technol..

[33] Ainur D., Chen Q., Sha T., Zarak M., Dong Z., Guo W., Zhang Z., Dina K., An T. (2023). Outdoor Health Risk of Atmospheric Particulate Matter at Night in Xi’an, Northwestern China. Environ. Sci. Technol..

[34] Zhao Z., Cao J., Shen Z., Huang R.-J., Hu T., Wang P., Zhang T., Liu S. (2015). Chemical Composition of PM_2.5_ at a High–Altitude Regional Background Site over Northeast of Tibet Plateau. Atmos. Pollut. Res..

[35] Cong Z., Kang S., Kawamura K., Liu B., Wan X., Wang Z., Gao S., Fu P. (2015). Carbonaceous Aerosols on the South Edge of the Tibetan Plateau: Concentrations, Seasonality and Sources. Atmos. Chem. Phys..

[36] Wan X., Kang S., Wang Y., Xin J., Liu B., Guo Y., Wen T., Zhang G., Cong Z. (2015). Size Distribution of Carbonaceous Aerosols at a High-Altitude Site on the Central Tibetan Plateau (Nam Co Station, 4730 m a.s.l.). Atmos. Res..

[37] Niu H., Kang S., Wang H., Zhang R., Lu X., Qian Y., Paudyal R., Wang S., Shi X., Yan X. (2018). Seasonal Variation and Light Absorption Property of Carbonaceous Aerosol in a Typical Glacier Region of the Southeastern Tibetan Plateau. Atmos. Chem. Phys..

[38] Xu J.-S., Xu M.-X., Snape C., He J., Behera S.N., Xu H.-H., Ji D.-S., Wang C.-J., Yu H., Xiao H. (2017). Temporal and Spatial Variation in Major Ion Chemistry and Source Identification of Secondary Inorganic Aerosols in Northern Zhejiang Province, China. Chemosphere.

[39] Xu M., Liu Z., Hu B., Yan G., Zou J., Zhao S., Zhou J., Liu X., Zheng X., Zhang X. (2022). Chemical Characterization and Source Identification of PM_2.5_ in Luoyang after the Clean Air Actions. J. Environ. Sci..

[40] Ostro B., Roth L., Malig B., Marty M. (2009). The Effects of Fine Particle Components on Respiratory Hospital Admissions in Children. Environ. Health Perspect..

[41] Huang T., Chen J., Zhao W., Cheng J., Cheng S. (2016). Seasonal Variations and Correlation Analysis of Water-Soluble Inorganic Ions in PM_2.5_ in Wuhan, 2013. Atmosphere.

[42] Yuan Q., Yang L., Dong C., Yan C., Meng C., Sui X., Wang W. (2014). Temporal Variations, Acidity, and Transport Patterns of PM_2.5_ Ionic Components at a Background Site in the Yellow River Delta, China. Air Qual. Atmos. Health.

[43] Zhao Z., Luo X.S., Jing Y., Li H., Pang Y., Wu L., Chen Q., Jin L. (2020). In Vitro Assessments of Bioaccessibility and Bioavailability of PM_2.5_ Trace Metals in Respiratory and Digestive Systems and Their Oxidative Potential. J. Hazard. Mater..

[44] Rahmatinia T., Kermani M., Farzadkia M., Nicknam M.H., Soleimanifar N., Mohebbi B., Jafari A.J., Shahsavani A., Fanaei F. (2021). Potential Cytotoxicity of PM_2.5_-Bound PAHs and Toxic Metals Collected from Areas with Different Traffic Densities on Human Lung Epithelial Cells (A549). J. Environ. Health Sci. Eng..

[45] Pang Y. (2021). Study on the Composition and Toxic Effects of Inhalable Particulates from Typical Motor Vehicle Exhaust Emissions. Master’s Thesis.

[46] Long Y.-M., Yang X.-Z., Yang Q.-Q., Clermont A.C., Yin Y.-G., Liu G.-L., Hu L.-G., Liu Q., Zhou Q.-F., Liu Q.S. (2020). PM_2.5_ Induces Vascular Permeability Increase through Activating MAPK/ERK Signaling Pathway and ROS Generation. J. Hazard. Mater..

[47] Chen Y., Luo X.-S., Zhao Z., Chen Q., Wu D., Sun X., Wu L., Jin L. (2018). Summer–Winter Differences of PM_2.5_ Toxicity to Human Alveolar Epithelial Cells (A549) and the Roles of Transition Metals. Ecotoxicol. Environ. Saf..

[48] Cachon B.F., Firmin S., Verdin A., Ayi-Fanou L., Billet S., Cazier F., Martin P.J., Aissi F., Courcot D., Sanni A. (2014). Proinflammatory Effects and Oxidative Stress within Human Bronchial Epithelial Cells Exposed to Atmospheric Particulate Matter (PM_2.5_ and PM>_2.5_) Collected from Cotonou, Benin. Environ. Pollut..

[49] Amouei Torkmahalleh M., Hopke P.K., Broomandi P., Naseri M., Abdrakhmanov T., Ishanov A., Kim J., Shah D., Kumar P. (2020). Exposure to Particulate Matter and Gaseous Pollutants during Cab Commuting in Nur-Sultan City of Kazakhstan. Atmos. Pollut. Res..

[50] Li H., Tang M., Luo X., Li W., Pang Y., Huang W., Zhao Z., Wei Y., Long T., Mehmood T. (2023). Compositional Characteristics and Toxicological Responses of Human Lung Epithelial Cells to Inhalable Particles (PM_10_) from Ten Typical Biomass Fuel Combustions. Particuology.

[51] Akhtar U.S., Rastogi N., McWhinney R.D., Urch B., Chow C.-W., Evans G.J., Scott J.A. (2014). The Combined Effects of Physicochemical Properties of Size-Fractionated Ambient Particulate Matter on in Vitro Toxicity in Human A549 Lung Epithelial Cells. Toxicol. Rep..

[52] Xu Y., Wang W., Zhou J., Chen M., Huang X., Zhu Y., Xie X., Li W., Zhang Y., Kan H. (2019). Metabolomics Analysis of a Mouse Model for Chronic Exposure to Ambient PM_2.5_. Environ. Pollut..

[53] Pniewska E., Sokolowska M., Kupryś-Lipińska I., Przybek M., Kuna P., Pawliczak R. (2014). The Step Further to Understand the Role of Cytosolic Phospholipase A2 Alpha and Group X Secretory Phospholipase A2 in Allergic Inflammation: Pilot Study. Biomed Res. Int..

[54] Maceyka M., Spiegel S. (2014). Sphingolipid Metabolites in Inflammatory Disease. Nature.

[55] Diaz-Vivancos P., de Simone A., Kiddle G., Foyer C.H. (2015). Glutathione—Linking Cell Proliferation to Oxidative Stress. Free. Radic. Bio. Med..

[56] Radi R. (2018). Oxygen Radicals, Nitric Oxide, and Peroxynitrite: Redox Pathways in Molecular Medicine. Proc. Natl. Acad. Sci. USA.

[57] Huang D., Zou Y., Abbas A., Dai B. (2018). Nuclear Magnetic Resonance-Based Metabolomic Investigation Reveals Metabolic Perturbations in PM_2.5_-Treated A549 Cells. Environ. Sci. Pollut. Res. Int..

[58] Moro J., Tomé D., Schmidely P., Demersay T.-C., Azzout-Marniche D. (2020). Histidine: A Systematic Review on Metabolism and Physiological Effects in Human and Different Animal Species. Nutrients.

[59] Du X., Zeng X., Pan K., Zhang J., Song L., Zhou J., Chen R., Xie Y., Sun Q., Zhao J. (2020). Metabolomics Analysis of Urine from Healthy Wild Type Mice Exposed to Ambient PM_2.5_. Sci. Total Environ..

[60] Wang X., Jiang S., Liu Y., Du X., Zhang W., Zhang J., Shen H. (2017). Comprehensive Pulmonary Metabolome Responses to Intratracheal Instillation of Airborne Fine Particulate Matter in Rats. Sci. Total Environ..

[61] Zhao C., Zhu L., Li R., Wang H., Cai Z. (2019). Omics Approach Reveals Metabolic Disorders Associated with the Cytotoxicity of Airborne Particulate Matter in Human Lung Carcinoma Cells. Environ. Pollut..

[62] Losito I., Patruno R., Conte E., Cataldi T.R.I., Megli F.M., Palmisano F. (2013). Phospholipidomics of Human Blood Microparticles. Anal. Chem..

[63] Lazarowski E.R., Boucher R.C. (2009). Purinergic Receptors in Airway Epithelia. Curr. Opin. Pharmacol..

