# Supplementary material for: Characterization of the Chemical Composition, Cytotoxicity, and Metabolomic Effects of PM2.5 in a Plateau City, China
Source: Toxics. 2025 Aug 29;13(9):729. doi: 10.3390/toxics13090729 (PMC12474249; doi:10.3390/toxics13090729)
Supplement: Supplementary file 1 [file toxics-13-00729-s001.zip › toxics-3789136-supplementary.pdf]

Supplementary information

for

Characterization of the chemical composition, cytotoxicity and metabolomic effects of $PM_{2.5}$ in a plateau city, China

Mengying Li [1], Lijuan Qi [1,*], Xinyi Xu[1], Rong Zhao [1], Xiaotong Wang [2], Yanhui Ha [1], Zhe Lin [1], Sujin Lu [1], Rong Chen [1] and Junchao Zhao [3]

[1] College of Ecological and Environmental Engineering, Qinghai University, Xining 810016, China; 18997169373@163.com (M.L.)

[2] Key Laboratory of Beijing on Regional Air Pollution Control, Beijing University of Technology, Beijing 100124, China

[3] Key Laboratory of Vehicle Emission Control and Simulation of Ministry of Ecology and Environment, Vehicle Emission Control Center, Chinese Research Academy of Environmental Sciences, Beijing 100124, China

* Correspondence: qhqilijuan@qhu.edu.cn.

Session S1 Pre-experiments for the concentrations of $PM_{2.5}$ suspensions

Prior to conducting in vitro cell experiments, exposure solutions with concentrations of 20, 40, 80, 160, and 240 mg/L were prepared for cell viability pre-experiments. The optimal

concentration was selected for subsequent experiments, with the research results exemplified by summer samples. The results are shown in Figure S1.The pre-experiment results indicated that cells exhibited a proliferative phenomenon at low concentrations, while the survival rate decreased significantly at 160 mg/L and 240 mg/L. Since the cell survival rate of the summer nighttime background site samples was above 90% at concentrations below 240 mg/L, the exposure concentration of 240 mg/L was chosen for in vitro toxicity experiments and subsequent metabolomics experiments.

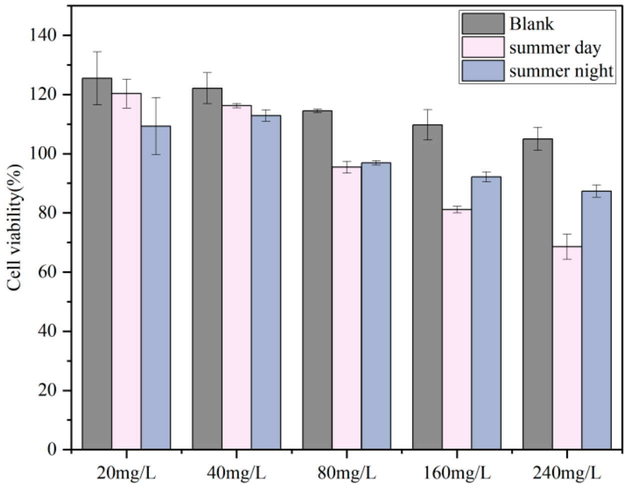

Figure S1. Changes in cell viability at different concentrations.

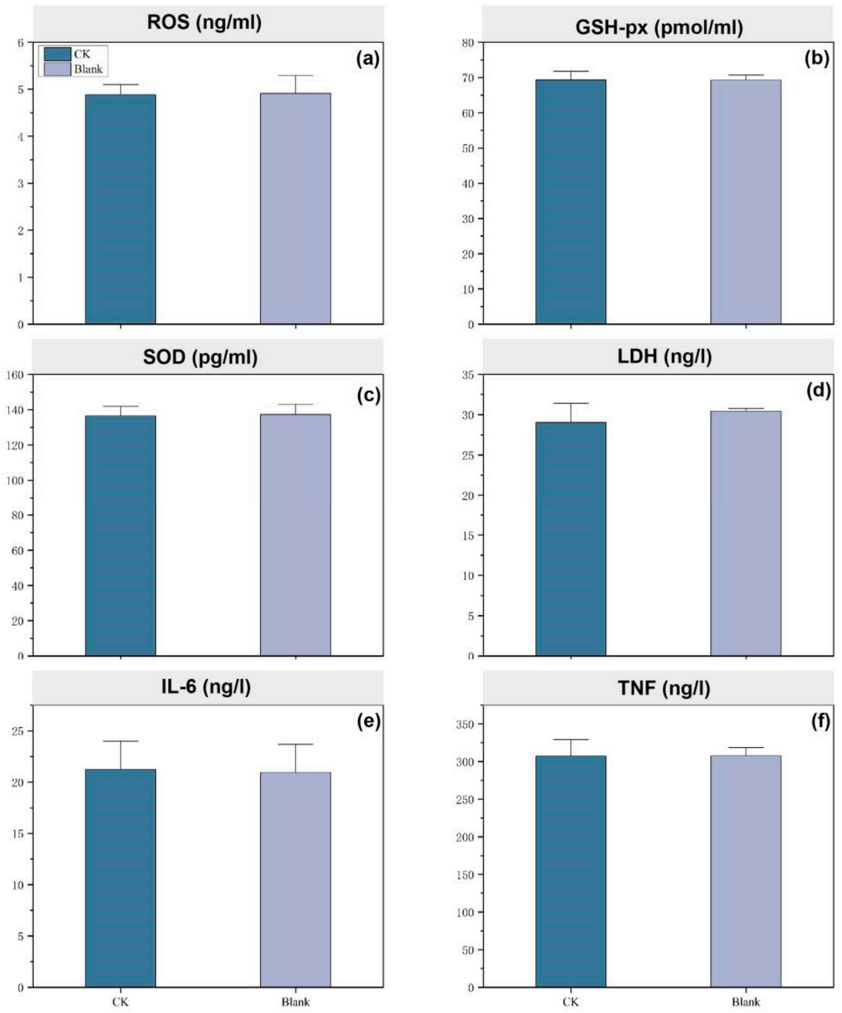

Figure S2. Results of in vitro cytotoxicity for blaank experiments

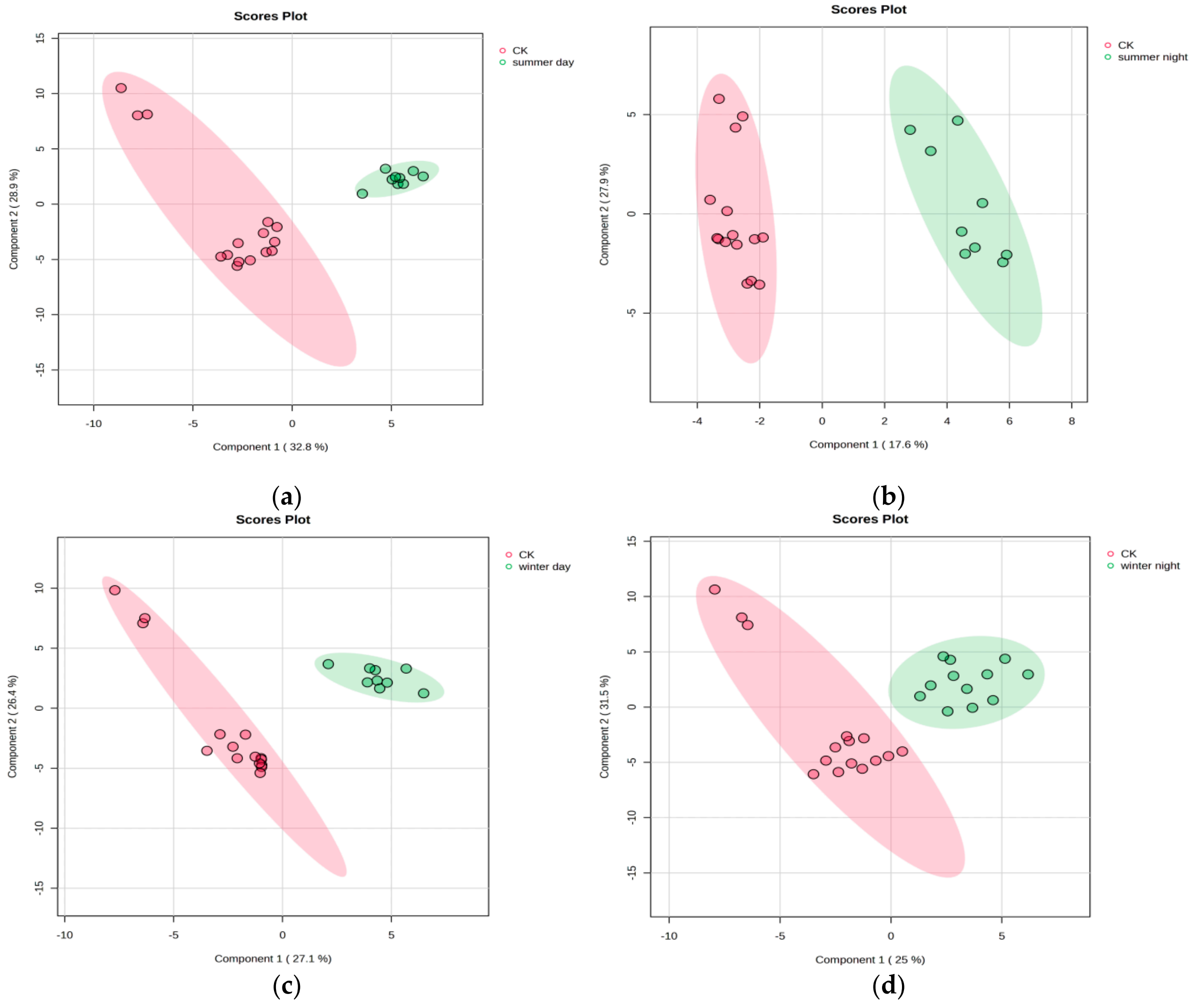

Figure S3. PLS-DA scatterplot (a) summer day; (d) summer night; (c) winter day; (d) winter night.
